# Pharmacokinetic/pharmacodynamic modeling and simulation of dotinurad, a novel uricosuric agent, in healthy volunteers

**DOI:** 10.1002/prp2.533

**Published:** 2019-11-26

**Authors:** Keisuke Motoki, Takako Igarashi, Koichi Omura, Hiroshi Nakatani, Takashi Iwanaga, Ikumi Tamai, Tetsuo Ohashi

**Affiliations:** ^1^ FUJI YAKUHIN CO., LTD. Saitama Japan; ^2^ Department of Research, Clinical Trial Center Kitasato University Kitasato Institute Hospital Tokyo Japan; ^3^ Faculty of Pharmaceutical Sciences Institute of Medical, Pharmaceutical and Health Sciences Kanazawa University Kanazawa Japan

**Keywords:** dotinurad, PK/PD model, URAT1, uric acid, uricosuric agent

## Abstract

This study aimed to investigate the pharmacokinetic and pharmacodynamic (PK/PD) profiles of dotinurad, a novel uricosuric agent, and to construct a PK/PD model to predict serum urate (SUA) levels after dotinurad administration in healthy men. PK/PD model was constructed using single‐dose study data considering the physiological features of urate handling. Model validation was performed by comparing the predicted SUA levels with the SUA levels in a multiple‐dose study. Dotinurad was absorbed rapidly, and its exposure increased proportionally in the tested dose ranges (0.5–20 mg) after a single‐dose administration. The PK model after oral administration was described using a one‐compartment model with first‐order absorption. Effects on SUA and renal urate excretion of dotinurad increased with dose escalation but were apparently saturable at a dose >5 mg. The simple maximal effect (*E*_max_) model was selected as the PD model of dotinurad on renal urate reabsorption, resulting in an estimated *E*_max_ of 0.51. The plasma concentration at the half‐maximal effect of dotinurad was 196 ng/mL. Other PD parameters were calculated from the change in SUA level or urinary excretion of urate before and after dotinurad administration. The predicted SUA levels, using the PK/PD model, were well‐fitted with the observed values. The constructed PK/PD model of dotinurad appropriately described the profiles of dotinurad plasma concentrations and SUA level in multiple administration study.

**Abbreviations**
%CV: coefficient of variation
AUC: area under the plasma concentration‐time curve
AUEC: area under the effect curve
CKD: chronic kidney disease
CLGut_UA_: non‐renal clearance of urate
CLR_UA_: renal clearance for urate
*C*_max_: maximum observed plasma concentration
Cu_UA_: urate concentration in urine
GFR: glomerular filtration rate
MAD: multiple ascending dose
PD: pharmacodynamic(s)
PK: pharmacokinetic(s)
SAD: single ascending dose
SUA: serum urate
*t*_1__/2_: terminal phase elimination half‐life
*T*_max_: time to *C*_max_
ULT: urate‐lowering therapy
Vd_UA_: distribution volume of urate
XOI: XOR inhibitor(s)
XOR: xanthine oxidoreductase
Xu_UA_: total urinary excretion amount of urate

## 1 INTRODUCTION

Gout is the most common inflammatory arthritis caused by the deposition of monosodium urate crystals within the joints and around tissues due to chronic hyperuricemia.^1^ A recent meta‐analysis suggested that hyperuricemia is an independent risk factor of multiple metabolic syndromes, such as hypertension, chronic heart disease, chronic kidney disease (CKD), and type 2 diabetes mellitus.^2^, ^3^, ^4^, ^5^ However, it is not clear if hyperuricemia is a cause or a result of these diseases.

In humans, uric acid (urate) is formed as an end product of purine metabolism by catalyzing enzyme xanthine oxidoreductase (XOR). Approximately two‐thirds of the daily turnover of urate is accounted for by renal excretion, with the remaining one‐third being excreted by non‐renal excretion, mainly into the gut as feces.^6^ The serum urate (SUA) level is determined by the balance between biosynthesis and elimination of urate in normal condition, that is, 4‐6 mg/dL (240‐360 µmol/L), whereas SUA level in hyperuricemia exceeds 7 mg/dL (>420 µmol/L) due to the overproduction and/or inefficient renal excretion of urate.^6^

For the treatment of pain and inflammation associated with acute flares, urate‐lowering therapy (ULT) has been adopted as a pharmacological treatment for the prevention of acute gout flares in patients with gout. Several guidelines for the management of gout recommend that SUA level should be lowered sufficiently to improve the signs and symptoms, that is, <6 mg/dL as a minimum target and often <5 mg/dL.^7^, ^8^, ^9^, ^10^ Clinically, the available drugs for ULT are XOR inhibitors (XOI) and uricosuric agents. Most guidelines recommend the use of XOI, such as allopurinol, febuxostat, and topiroxostat (labeled only in Japan), as first‐line ULT medications. These drugs lower SUA level effectively in most cases; however, many patients do not achieve the recommended SUA level with XOI monotherapy.^11^ In such cases, guidelines recommend that an alternative or combination therapy of XOI with a uricosuric agent, such as benzbromarone or probenecid, should be considered. However, in 2003, benzbromarone was withdrawn from the market in many countries because of its risk of severe hepatotoxicity.^12^ Although probenecid was approved in many countries such as USA, its widespread use is also limited due to its ineffectiveness, potential nephrotoxicity and significant drug‐drug interaction potential with widely used drugs such as nonsteroidal anti‐inflammatory drugs and antibiotics. Accordingly, a novel, safer and accessible uricosuric agent is required.

Dotinurad, also called as FYU‐981, is a novel Selective Uric Acid Reabsorption Inhibitor (SURI) that shows potent inhibitory effects on the uptake of urate in human renal brush‐border membrane vesicles.^13^ In 90% of patients with gout, hyperuricemia results from reduced renal urate excretion^14^; thus, uricosuric agents should effectively lower the level of SUA. However, excessive amounts of renal urate excretion is associated with a higher risk of nephrolithiasis.^15^ In addition, excessive urate‐lowering effects possibly cause the progression of various neurodegenerative disorders, such as Parkinson's disease,^16^ Alzheimer's disease^17^ and amyotrophic lateral sclerosis.^18^ Given these possibilities, it is important to control SUA levels and renal excretion of urates within an adequate range during ULT, especially with uricosurics. It is important to construct a PK/PD model in order to maintain SUA level within an appropriate range. However, it is unknown if urate handling in patients differs from that in healthy people. In this study, we aimed to construct the PK/PD model of dotinurad and to predict SUA levels in healthy volunteers using phase 1 clinical study of dotinurad. Firstly, the pharmacokinetic (PK) and pharmacodynamic (PD) profiles of dotinurad in healthy male volunteers were evaluated. Secondly, a PK/PD model of dotinurad was constructed using derived data from the single ascending dose (SAD) study to predict SUA profiles in the multiple ascending dose (MAD) study. The models were assessed by comparing the predicted and observed values of dotinurad in the MAD study.

## 2 MATERIALS AND METHODS

### 2.1 Subjects

For the SAD study, a total of 54 subjects were enrolled, and they all completed the study (ClinicalTrials.gov identifier:. NCT02348307) except for one subject who dropped out due to domestic reason; for the MAD study, 18 subjects were enrolled, and all completed the study (NCT02348333). Subject demographics were comparable in both groups. All the subjects were healthy Japanese men (age range, 20‐35 years; body mass index, ≥18.5 and <25.0 kg/m^2^) for both studies.

These studies were conducted at an institute hospital in Japan. The study protocol was conducted in accordance with the Declaration of Helsinki, the standards of the Japanese Pharmaceutical Affairs Law, and International Conference on Harmonization and Good Clinical Practice guidelines. The study protocols were approved by the institutional review board of the institute, and all subjects provided written informed consent to participate prior to the initiation of each study.

### 2.2 Study design and sample collection

The SAD and MAD studies were randomized, double‐blind, placebo‐controlled, dose escalation, and parallel group comparison studies. In the SAD study, nine fasted healthy subjects were randomly assigned in a 2:1 ratio to receive either a single oral dose of 0.5, 1, 2, 5, 10 and 20 mg of dotinurad (n = 6) or placebo (n = 3). From the point of ethical consideration, the number of subjects in the placebo group was set at the half of the dotinurad treated groups (n = 3) as the minimum number of subjects that allows comparison of safety during hospitalization in each dotinurad treated group. Placebo formulation was composed of more proportion of lactose hydrate instead of dotinurad. The effect of food on the PK of dotinurad was evaluated at 5‐mg dose with the same subjects in the fasted dosing study. In the MAD study, nine fed healthy subjects received either 2 or 5 mg of dotinurad (n = 6) or placebo (n = 3) once daily over 7 days.

In the SAD study, blood samples were collected at 0 (30 min before dosing), 0.25, 0.5, 1, 1.5, 2, 4, 6, 8, 12, 24, 36, and 48 hours after dosing to determine the plasma dotinurad concentration as well as SUA and serum creatinine concentrations. In the MAD study, blood samples were collected at 0 (only at day 0), 0.25, 0.5, 1, 1.5, 2, 4, 6, 8, 12, and 24 hours after dosing in days 0, 3, 6, and only at 12 and 24 hours in days 1, 2, 4, 5, and additionally at 36, 48, and 72 hours in day 6. Urine samples were collected for 24 hours before dosing and 0‐6, 6‐12, 12‐24, 24‐36, and 36‐48 hours after dosing in the SAD study, or every 24 hours before and after dosing, and up to 72 hours only after the last dose in the MAD study, to determine dotinurad, urate, and creatinine concentrations in the urine.

### 2.3 Sample analysis

Plasma and urine dotinurad concentrations were analyzed using high‐performance liquid chromatography with tandem mass spectrometry using the validation method at FUJI YAKUHIN CO., LTD. Sample clean‐up was achieved by solid phase extraction in a 96‐well plate. The concentration ranges of the calibration curves of dotinurad were 1‐1000 ng/mL and 10‐1000 ng/mL for the plasma and urine analyses respectively. All the calibration curves had *r* values >0.9974. The pre‐established acceptance criteria of calibration standard and quality control samples were within ±15% of their theoretical concentrations with coefficient of variation (%CV) of <15% (only the criterion of the lower limit of quantitation of the samples was 20%), and all analyses met this criteria. Samples with concentrations above the upper limit of quantitation were diluted with blank matrix and reanalyzed. The plasma and urine samples were stored at −30°C, and all the samples were analyzed within the validated storage stability duration. The concentrations of urate and creatinine in the serum and urine were measured by enzymatic methods for conventional clinical test.

### 2.4 PK analysis

Plasma PK parameters of dotinurad were derived using Phoenix WinNonlin (version 6.2, Pharsight Corporation as part of Certara, St. Louis, MO). Plasma PK parameters included maximum observed plasma concentration (*C*_max_), time to *C*_max_ (*T*_max_), area under the plasma concentration‐time curve (AUC) from time 0 to 24 hour (AUC_0–24_), time 0 to infinity (AUC_0–inf_), and terminal phase elimination half‐life (*t*_1/2_). AUC was calculated using the linear trapezoidal rule, and the apparent oral clearance (CL/F) was determined. The accumulation ratio (R_AUC0–24_) was calculated as the ratio of AUC_0–24_ on day 4 or day 7 to AUC_0–24_ on day 1.

### 2.5 PD analysis

The area under the effect curve (AUEC) was calculated as the AUC of the SUA‐time curve. The total urinary excretion amount of urate (Xu_UA_) was calculated from the urate concentration in urine (Cu_UA_) and urine volume during the sampling time. The renal clearance for urate (CLR_UA_) was calculated as Xu_UA_/AUEC.

### 2.6 PK/PD modeling of dotinurad

Based on the observed mono‐exponential decline of the plasma concentrations of dotinurad, the %CV of estimated parameters, and the model comparison analysis using Akaike's information criterion, a one‐compartment model with first‐order absorption was selected as the PK model of dotinurad.

**Figure 1:**
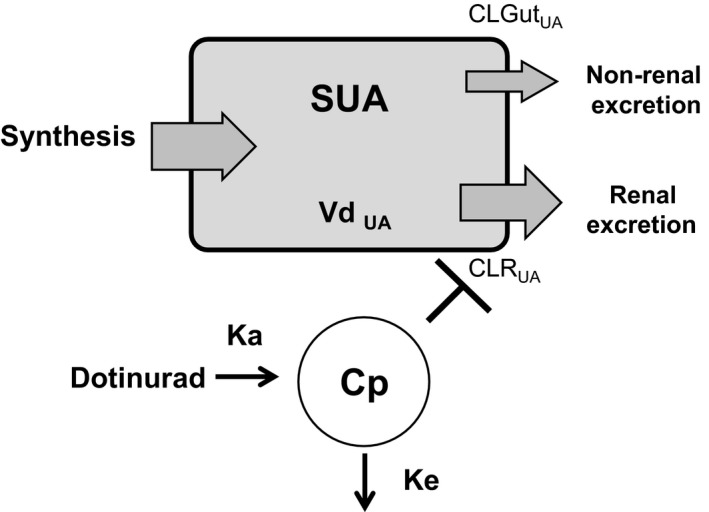
Structural pharmacokinetic/pharmacodynamic model diagram of dotinurad on the renal clearance of urate

To predict the SUA level after dotinurad administration in the MAD study, a simple PD model of dotinurad on urate clearance was constructed based on the physiological features of urate handling in the serum and urine.^6^ Given the uricosuric effect of dotinurad (see Results), the scheme of the PD model of dotinurad is shown in Figure 1.

This model was constructed under the following assumptions:

1. Before dotinurad administration, the amount of urate production (shown as “Synthesis,” involving the absorption of purine body from food intake) is a constant value identical to the sum of the renal and non‐renal (mainly into feces from the gut) urate excretion.
2. The ratio of the renal to non‐renal urate excretion is 2:1 in the normal state, that is, before the administration of dotinurad.
3. Dotinurad affects only CLR_UA_ and does not affect non‐renal clearance of urate (CLGut_UA_).
4. The distribution volume of urate (Vd_UA_) is constant.
5. Dotinurad affects CLR_UA_ via the inhibition of urate reabsorption in a competitive and plasma concentration‐dependent manner.
6. The glomerular filtration rate (GFR) is not affected by dotinurad.

Under these assumptions, SUA and CLR_UA_ in a physiological condition can be described by the following equations:

(1) $$\text{SUA}=\left\{\text{Synthesis}-\left({\text{Xu}}_{\text{UA}}+{\text{Xf}}_{\text{UA}}\right)+{\text{UA}}_{\text{pool}}\right\}/{\text{Vd}}_{\text{UA}}$$

(2) $${\text{Xu}}_{\text{UA}}={\text{CLR}}_{\text{UA}}\times \text{AUEC}$$

(3) $${\text{Xf}}_{\text{UA}}={\text{CLGut}}_{\text{UA}}\times \text{AUEC}$$

UA_pool_ is the baseline value of the total amount of urate in the body. Xf_UA_ is the total amount of urate excretion in the feces. Vd_UA_ was estimated from changes in SUA level before and after dotinurad dosing (∆SUA) and Equation (1).

(4) $${\text{Vd}}_{\text{UA}}=\left(\Delta {\text{Xu}}_{\text{UA}}+\Delta {\text{Xf}}_{\text{UA}}\right)/\Delta \text{SUA}$$

CLR_UA_ and AUEC before and after the administration of dotinurad were calculated as the mean or total value during 24 hours respectively. ∆SUA was the difference between SUA at 0 and that at 24 hours after the administration of dotinurad. ∆Xu_UA_ and ∆Xf_UA_ were calculated from the difference of Xu_UA_ or Xf_UA_ for 24 hours between before and after the administration of dotinurad. The baseline values of Xf_UA_, CLGut_UA_ and “Synthesis” were estimated from Xu_UA_ and CLR_UA_ before the administration of dotinurad using the following equations:

(5) $${\text{Xf}}_{\text{UA}}=\frac{1}{2}\times {\text{Xu}}_{\text{UA}}$$

(6) $${\text{CLGut}}_{\text{UA}}=\frac{1}{2}\times {\text{CLR}}_{\text{UA}}$$

(7) $$\text{Synthesis}={\text{Xu}}_{\text{UA}}+{\text{Xf}}_{\text{UA}}=\frac{3}{2}\times {\text{Xu}}_{\text{UA}}$$

Equation (5) was derived from the assumption (2). Equation (6) was derived from Equations (2), (3) and (5). Equation (7) was derived from assumption (1) and Equation (5). From assumption (3), Xf_UA_ after the administration of dotinurad was calculated as the product of SUA and estimated CLGut_UA_. Vd_UA_ was estimated as the mean value of all the subjects who received each dose of dotinurad (n = 35).

The effect of dotinurad on CLR_UA_ was estimated using the following equations:

(8) $${\text{CLR}}_{\text{UA}}=\text{GFR}-f_{\text{reabs}}\times \text{GFR}\times \left(1-\frac{E_{\max}\times \text{Cp}}{{\text{EC}}_{50}+\text{Cp}}\right)$$

In this study, creatinine clearance (Ccr), adjusted to body surface area of 1.73 m^2^, was used as GFR. The fractional reabsorption of filtered urate (f_reabs_) was calculated from the CLR_UA_ during 24 hours in subjects who received placebo (n = 18) based on the following equation:

(9) $${\text{CLR}}_{\text{UA}}=\text{GFR}-f_{\text{reabs}}\times \text{GFR}$$

*E*_max_ and EC_50_ represent the maximum effect of dotinurad on CLR_UA_ and the plasma concentration of dotinurad which achieves the half‐maximum effect respectively. Cp represents the mean plasma concentration of dotinurad during the evaluation of CLR_UA_. *E*_max_ and EC_50_ were obtained by fitting the plot of calculated {*E*_max_　×Cp/(EC_50_ + Cp)} vs Cp to simple *E*_max_ model using Phoenix WinNonlin. CLR_UA_ and Cp were calculated as the mean value during 0‐6, 6‐12, 12‐24 and 24‐48 hours in each subject who received each dose of dotinurad.

### 2.7 Model simulation

Using the fixed PK model of dotinurad and estimated PD parameters, the SUA–time profile was predicted after the multiple administrations of dotinurad. The mean observed baseline SUA and CLR_UA_ were used as initial values. Additionally, the initial UA_pool_ was calculated using Equation (1). CLR_UA_ was estimated every hour, and Xu_UA_ and Xf_UA_ during −24 to 0 hours, 0‐24 hours and 24‐48 hours were estimated using the SUA and CLR_UA_ or CLGut_UA_ values before 1 hour of dotinurad administration. Finally, SUA level was estimated from Equation (1), using “Synthesis” in the scheme and Vd_UA_ as the constant value. Model confirmation was performed by comparing the predicted SUA–time curve with the observed values in the MAD study.

### 2.8 Statistical analysis

Observed PK and PD parameters were summarized using descriptive statistics. Each datum was represented as the mean + or ±standard deviation (SD). Estimated PK and PD parameters were represented as the mean and %CV. SAS software (version 9.2, SAS Institute Inc, Cary, NC) was used to perform statistical analyses. Dose‐proportional assessment for *C*_max_ and AUC was performed using a power model as follows:

$$\text{Log}\left(\text{AUC}\right)\text{orLog}\left(C_{\max}\right)=a+b\times \text{Log}\left(\text{Dose}\right).$$

where slope b is close to unity, and the relationship between dose and PK parameters was concluded to be dose proportional within the dose range studied. The effect of food intake on PK parameters was analyzed by paired *t*‐test.

## 3 RESULTS

### 3.1 PK of dotinurad in healthy male volunteers

**Figure 2:**
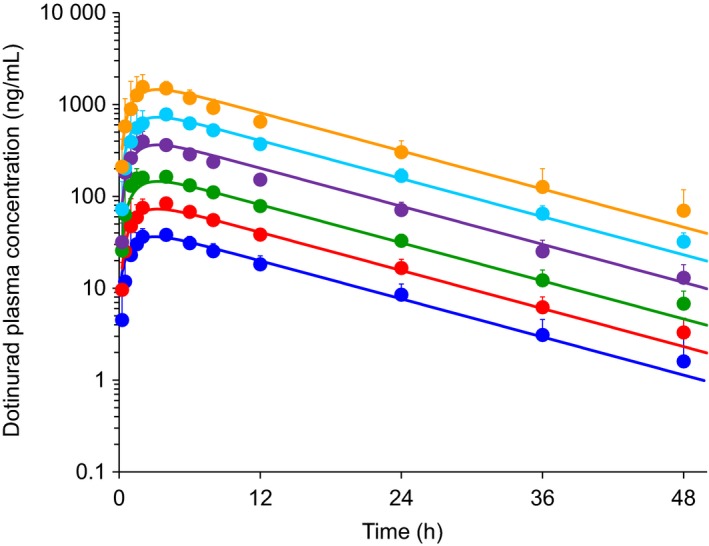
The observed (mean + SD) and predicted dotinurad plasma concentration‐time profiles after single‐dose administration of 0.5‐20 mg of dose of dotinurad in a fasted state. Each symbol represents the observed data points. Solid lines represent the predicted results obtained by constructed PK models. Blue, 0.5 mg; red, 1 mg; green, 2 mg; purple, 5 mg; sky blue, 10 mg; and orange, 20 mg

In the SAD study, plasma concentrations of dotinurad in fasted volunteers increased in a dose‐proportional manner (Figure 2, Table 1). *C*_max_ and AUC_0–inf_ values also increased dose proportionally from 0.5 mg up to 20 mg based on the power model analysis and the slope of the log‐transformed *C*_max_ and AUC_0–inf_ included 1.0 (90% confidence interval (CI): *C*_max_ 0.975‐1.049, AUC_0–inf_ 0.936‐1.016). *T*_max_, *t*_1/2_ and CL/F values were generally similar for all the doses, and the mean values were 2.77, 9.77 hours and 0.850 L/h respectively. After food intake, *C*_max_ and AUC_0–inf_ decreased significantly (*P* < .05); the geometric mean ratio (fed/fasted) of *C*_max_ was 0.84 (90% CI: 0.76‐0.93), and AUC_0–inf_ was 0.95 (90% CI: 0.92‐0.99). *T*_max_ was delayed by 1.67 hours (*P* < .05), but *t*_1/2_ was not changed.

**Table 1:** Mean pharmacokinetic parameters after single‐dose administrations of dotinurad

	Fasted						Fed
	0.5 mg (n = 6)	1 mg (n = 6)	2 mg (n = 5)	5 mg (n = 6)	10 mg (n = 6)	20 mg (n = 6)	5 mg (n = 6)
*C*_max_ (ng/mL)	41.5 ± 4.5	89.2 ± 10.8	175.2 ± 33.0	447.8 ± 72.6	858.2 ± 136.3	1783.6 ± 351.5	373.8 ± 40.7*
*t*_max_ (h)	2.67 ± 1.03	3.33 ± 1.03	3.10 ± 1.24	2.00 ± 1.10	3.25 ± 1.17	2.25 ± 1.41	3.67 ± 0.82*
*t*_1/2_ (h)	9.67 ± 1.77	9.60 ± 1.27	9.53 ± 1.11	9.27 ± 1.10	9.87 ± 0.83	10.65 ± 2.85	9.42 ± 1.03
AUC_0–inf_ (ng·h/mL)	613 ± 134	1276 ± 189	2599 ± 381	5526 ± 419	12 126 ± 1204	23 398 ± 7055	5270 ± 520*
CL/F (L/h)	0.848 ± 0.176	0.801 ± 0.144	0.783 ± 0.113	0.910 ± 0.077	0.831 ± 0.079	0.927 ± 0.291	0.957 ± 0.097*

Note

Data are shown as mean ± SD. Fed group was compared to same dose of fasted group (5 mg) (* indicates *P* < .05; paired *t*‐test).

Abbreviations: AUC_0–inf_, area under the plasma concentration‐time curve from time 0 to infinity; CL/F, apparent oral clearance; *C*_max_, maximum plasma concentration; SD, standard deviation; *t*_max_, time at the maximum plasma concentration; *t*_1/2_, terminal phase elimination half‐life.

In the MAD study, plasma concentration of dotinurad achieved a steady state at day 4, and the R_AUC0–24_ at days 4 and 7 were comparable, showing 1.21 and 1.22 at 2 mg dose, and 1.18 and 1.21 at 5 mg dose respectively (Table 2). Other PK parameters were comparable between day 4 and day 7 at each dose.

**Table 2:** Mean pharmacokinetic parameters after multiple dose administrations of dotinurad

	2 mg (n = 6)			5 mg (n = 6)		
	Day 1	Day 4	Day 7	Day 1	Day 4	Day 7
*C*_max_ (ng/mL)	188 ± 29.6	224 ± 35.0	228 ± 29.8	438 ± 55.1	493 ± 75.8	514 ± 106
*t*_max_ (h)	3.50 ± 0.55	3.67 ± 0.52	3.67 ± 0.52	4.17 ± 0.98	4.00 ± 1.10	3.83 ± 1.33
*t*_1/2_ (h)	10.95 ± 1.93	10.31 ± 2.04	9.73 ± 1.34	10.86 ± 1.53	10.56 ± 1.58	9.73 ± 1.11
AUC_0–24_ (ng·h/mL)	2195 ± 233	2649 ± 346	2680 ± 425	5211 ± 715	6164 ± 1081	6321 ± 1189
CL/F (L/h)	0.696 ± 0.103	0.609 ± 0.113	0.621 ± 0.127	0.732 ± 0.155	0.650 ± 0.156	0.660 ± 0.163
R_AUC0–24_	—	1.21 ± 0.10	1.22 ± 0.11	—	1.18 ± 0.08	1.21 ± 0.67

Note

Data are shown as the mean ± SD.

Abbreviations: AUC_0–24_, area under the plasma concentration‐time curve from time 0 to 24 hours; CL/F, apparent oral clearance;* C*_max_, maximum plasma concentration; R_AUC0–24_, accumulation ratio of AUC_0–24_ of the day against day 1; SD, standard deviation;* t*_1/2_, terminal phase elimination half‐life; *t*_max_, time at the maximum plasma concentration.

### 3.2 SUA‐lowering effect and uricosuric effect of dotinurad in the SAD study

**Figure 3:**
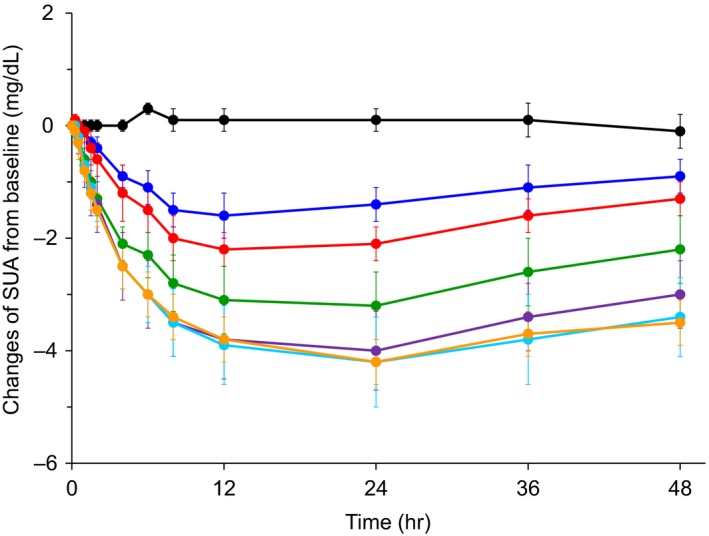
The changes in SUA (mean ± SD) after single‐dose administration of placebo or 0.5‐20 mg dose of dotinurad in a fasted state. Each symbol represents the observed data points. Gray, placebo; blue, 0.5 mg; red, 1 mg; green, 2 mg; purple, 5 mg; sky blue, 10 mg; and orange, 20 mg

The mean (SD) baseline SUA, Xu_UA,_ and CLR_UA_ values of all the subjects were 5.71 (0.75) mg/dL, 575 (100) mg/day, and 7.10 (1.51) mL/min (data not shown) respectively. Dotinurad decreased the SUA level until 48 hours (Figure 3). The maximal decrease at this time point was 1.6‐4.2 mg/dL from the baseline at each dose. A saturable SUA‐lowering effect of dotinurad was observed, and the effect almost reached the maximum at 5‐mg dose. The Xu_UA_ during 0‐24 hour after the administration of dotinurad was increased from the baseline at all the doses, ranging from 869 to 1458 mg, and, then, returned to baseline during 24‐48 hour. CLR_UA_ during 0‐24 hour after the administration was also increased, ranging from 12.1 to 47.9 mL/min. Its increase from the baseline was still observed during 24‐48 hour at doses >2 mg. Similar to the SUA‐lowering effect, the increasing effect of dotinurad on Xu_UA_ and CLR_UA_ seemed saturable, reaching almost maximum at 5‐mg dose.

### 3.3 PK/PD parameter estimation and prediction of SUA level after the multiple administrations of dotinurad

Table 3 presents estimated PK/PD model parameters. For PK parameters, the uncertainty %CV was low (<14.2). Additionally, at 5‐mg dose, plasma dotinurad in the fed state was well predictable from the parameters estimated from the data in the fasted state. Finally, the predicted plasma concentrations of dotinurad after multiple dose administrations were close to the observed values in the MAD study.

**Table 3:** Estimated pharmacokinetic pharmacodynamic model parameters

Pharmacokinetic parameters	Estimate (%CV)
V/F (mL)	10 585 (7.2)
Ka (/h)	0.770 (14.2)
Ke (/h)	0.0795 (9.3)
Vd_UA_ (L)	16.0 (36.6)
f_reabs_	0.943 (0.6)
EC_50_ (ng/mL)	196 (11.9)
*E*_max_	0.51 (4.6)

Abbreviations: CV, coefficient of variation; EC_50_, plasma concentration of dotinurad at half‐maximal effect; *E*_max_, maximal effect of dotinurad on renal urate clearance; f_reabs_, fractional reabsorption of urate from kidney; Ka, absorption rate constant of dotinurad; Ke, terminal phase elimination rate constant of dotinurad; Vd_UA_, distribution volume of urate; V/F, apparent distribution volume of dotinurad after oral administration.

For PD parameters, the mean value (%CV) of Vd_UA_ was estimated as 16.0 (36.6) L from Equation (4) and f_reabs_ as 0.943 (0.6) from Equation (9). The effect of dotinurad on CLR_UA_ were also estimated with 196 (11.9) ng/mL as the EC_50_ and 0.51 (4.6) as the *E*_max_.

**Figure 4:**
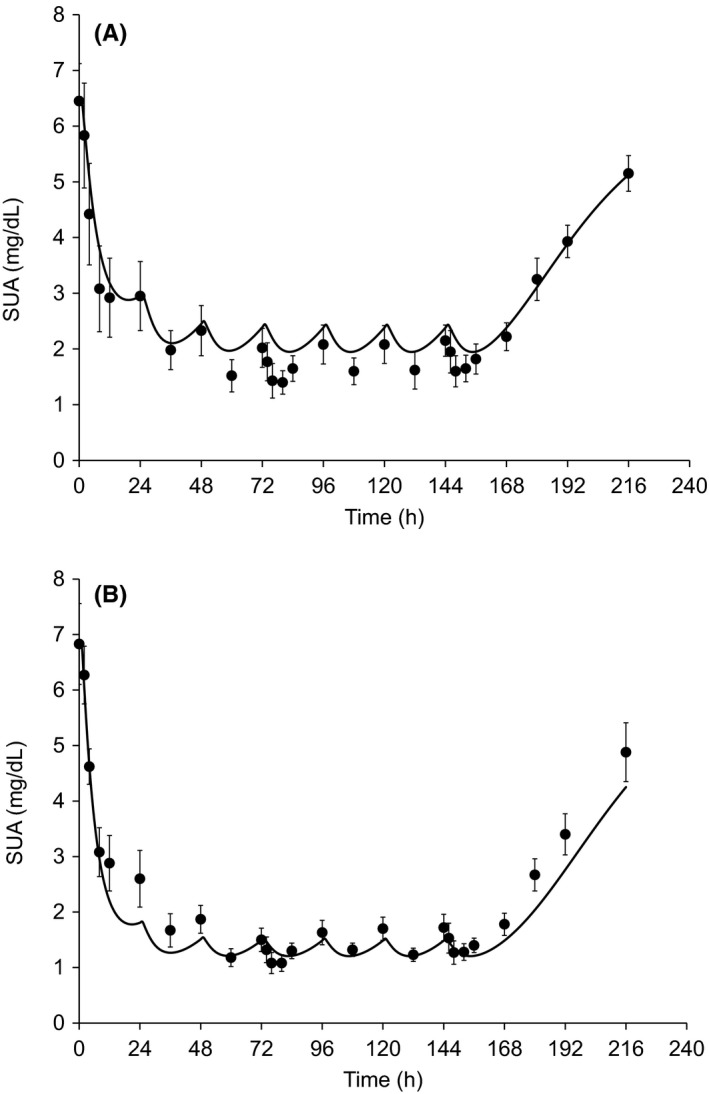
The observed (mean ± SD) and predicted SUA–time profiles after multiple dose administrations of 2‐mg (A) or 5‐mg (B) dose of dotinurad. Each symbol represents the observed data points. Solid lines represent the predicted value obtained by constructed pharmacokinetic and pharmacodynamic models of dotinurad

In this study, the observed and predicted SUA levels were compared, and the constructed PK/PD model was considered able to predict the observed SUA level after multiple dose administrations of dotinurad, although the predicted values were slightly underestimated at the 2‐mg dose (Figure 4).

## 4 DISCUSSION

This study evaluated the PK and PD profiles of single increasing doses of dotinurad, a novel uricosuric agent, in healthy male subjects. It also established a PK/PD model for the prediction of SUA levels after multiple administration of dotinurad, based on the results of a single administration study.

After oral administration under a fasted condition, dotinurad was absorbed quickly. It exhibited similar peak time and terminal phase elimination, and its plasma concentration was maintained during 24 hours at all the doses from 0.5 to 20 mg. The systemic exposure increased in a dose‐proportional manner. The changes in the value of *C*_max_ and AUC_0–inf_ of dotinurad were essentially negligible in the fed and fasted states, although statistical significance was observed between fed and fasted states. We constructed a PK model based on the data in a fasted condition. After multiple dose administrations, dotinurad did not show any accumulation. The plasma concentrations of dotinurad could be well‐predicted using the constructed PK model.

In a previous study on isolated human renal brush‐border membrane vesicle, the uricosuric potency of dotinurad was attributed to its inhibitory effect on the renal reabsorption of urate.^13^ Recently, several membrane transporters involved in renal urate handling have been identified,^19^ and one of such transporters is URAT1.^20^ URAT1 is involved primarily in the renal apical uptake of urate, and it is the primary target of most recent uricosuric drugs. In fact, dotinurad inhibited urate transport in a concentration‐dependent manner in human URAT1–overexpressed MDCKII cells (IC_50_ = 37.2 nmol/L) and exhibited little inhibitory effect on XOR activity in vitro.^21^ In the present study, after the single oral administration of dotinurad, SUA level was decreased rapidly, and the Xu_UA_ and CLR_UA_ were increased at all the tested doses. From these results, dotinurad effectively lowered SUA levels in human, and this effect was attributed to its uricosuric effect. In the SAD study, it is worthy of note that although the *C*_max_ and AUC of dotinurad, at up to 20‐mg dose, were increased dose proportionally, a decrease in SUA level and an increase in urinary urate excretion were almost maximum at the 5‐mg dose. Therefore, the uricosuric effect of dotinurad could be represented by a saturable and simple *E*_max_ model.

Estimated Vd_UA_ was assumed as constant; additionally, Vd_UA_ was estimated from the data of healthy volunteers in this study as 0.27 L/kg (calculated using the mean body weight of subjects in the SAD study, 60 kg). Scott et al reported urate pool size in humans by analyzing urinary urate excretion after the intravenous administration of radiolabeled urate solution.^22^ From the data in this report, the mean Vd_UA_ in the subjects with normal SUA values was calculated as 0.34 L/kg, which was obtained from the SUA values and the urate pool size in the body. Similarly, Scott et al also reported the urate pool size in patients with gout having tophi as 0.31 L/kg. Taken together, Vd_UA_ was similar in the subjects with and without gout; thus, the parameters estimated in this study could also be applied in patients with gout.

The effect of dotinurad on CLR_UA_ is expressed in Equation (8). The EC_50_ of dotinurad in human in vivo was calculated as 196 ng/mL (547 nmol/L) as total plasma concentration. In contrast, only the free fraction of plasma dotinurad is able to access the possible pharmacological target site, that is, the luminal side of the proximal tubules. The plasma protein binding rate of dotinurad was 99.3%, and the free concentration in the plasma at EC_50_ was 3.8 nmol/L. *E*_max_, another PD parameter of dotinurad, was 0.51. Interestingly, CLR_UA_ and fractional excretion of urate in patients with renal hypouricemia, mainly caused by loss‐of‐function homozygous mutation of SLC22A12/URAT1, increased to 68.3 ± 31.6 mL/min and 0.584 ± 0.264 respectively.^23^ However, in this study, CLR_UA_ and fractional excretion of urate were 47.9 mL/min and 0.51 respectively. Given the comparable results in the two studies, the uricosuric effect of dotinurad in human may possibly be caused by the inhibition of URAT1.

The constructed PK/PD model of dotinurad was able to reasonably predict the SUA‐time profiles after multiple administrations of dotinurad at 2 or 5 mg; however, the predicted SUA level at 2‐mg dose tended to be slightly underestimated compared with the observed value. EC_50_ and *E*_max_ were estimated based on saturable kinetic analysis; therefore, the influence of the variability of the estimated parameters was possibly more sensitive in the prediction at a low dose than at a high dose. Although there is a need for model improvement, our PD model has the advantage of flexibility. Patients with gout or hyperuricemia frequently have multiple comorbidities including CKD, and lower GFR leads to decreased urinary urate excretion and increased SUA level, both of which are strongly associated with gout.^24^ Our PD model includes GFR as a PD parameter, and hence, this model may be applied to predict SUA level in patients with renal dysfunction. Our PD model includes CLR_UA_ and “Synthesis,” as PD parameters. Although some modifications might be needed, this model could be applied to predict SUA level in each disease type of hyperuricemia, such as under‐excretion, overproduction or the combination of both, and in patients receiving a combination therapy of XOI and uricosuric agent. Future studies are needed to clarify if the PD model in this study can be applied to patients with gout/hyperuricemia.

Based on the simulation results, 0.5 mg or higher dose of dotinurad is expected to achieve the target SUA level. Additionally, the predicted SUA level after the administration of 5‐mg dose of dotinurad would maintain the SUA level below 3 mg/dL. Some studies have suggested that urate may have protective effects against various neurodegenerative disorders,^16^, ^17^ and most guidelines for the management of gout do not recommend keeping the SUA at a lower level for a long period.^7^, ^9^ From the present predicted results, 0.5‐2 mg dose of dotinurad is expected to be appropriate for pharmacological treatment and management of gout. These predictions were based on the assumptions that PK/PD modeling parameters in patients are not different from those in healthy subjects; thus, further investigations are needed to apply our PK/PD model to patients.

In conclusion, PK/PD profiles of dotinurad were evaluated in healthy human volunteers. The results of this study demonstrated that the SUA‐lowering effect of dotinurad was dependent on its uricosuric effect. The constructed PK/PD model in healthy subjects appropriately described the time profiles of dotinurad plasma concentrations and SUA levels after single and multiple dose administration. This indicated that the constructed PK/PD model well‐predicted the effects of uricosuric drug on SUA. Future studies are needed to determine if the constructed model can be applied to patients with gout or hyperuricemia or in special populations such as patients with renal impairment.

## DISCLOSURES

Dr Ikumi Tami serve as a consultant for FUJI YAKUHIN CO, LTD. This work was supported by FUJI YAKUHIN CO, LTD.

## AUTHOR CONTRIBUTIONS

Takako Igarashi and Koichi Omura analyzed plasma dotinurad concentration. Dr Hiroshi Nakatani was the principal investigator of the clinical studies. Keisuke Motoki, Takako Igarashi, and Takashi Iwanaga performed PK/PD model analysis. Keisuke Motoki wrote the manuscript. Dr Takashi Iwanaga and Dr Ikumi Tamai provided feedback on the manuscript. All authors read and approved the final manuscript.

## Data Availability

The data that support the findings of this study are available from the corresponding author upon reasonable request.
