# Supplementary figures and images for: Pharmacokinetic/pharmacodynamic modeling and simulation of dotinurad, a novel uricosuric agent, in healthy volunteers
Source: Pharmacol Res Perspect. 2019 Nov 26;7(6):e00533. doi: 10.1002/prp2.533 (PMC6880184; doi:10.1002/prp2.533)

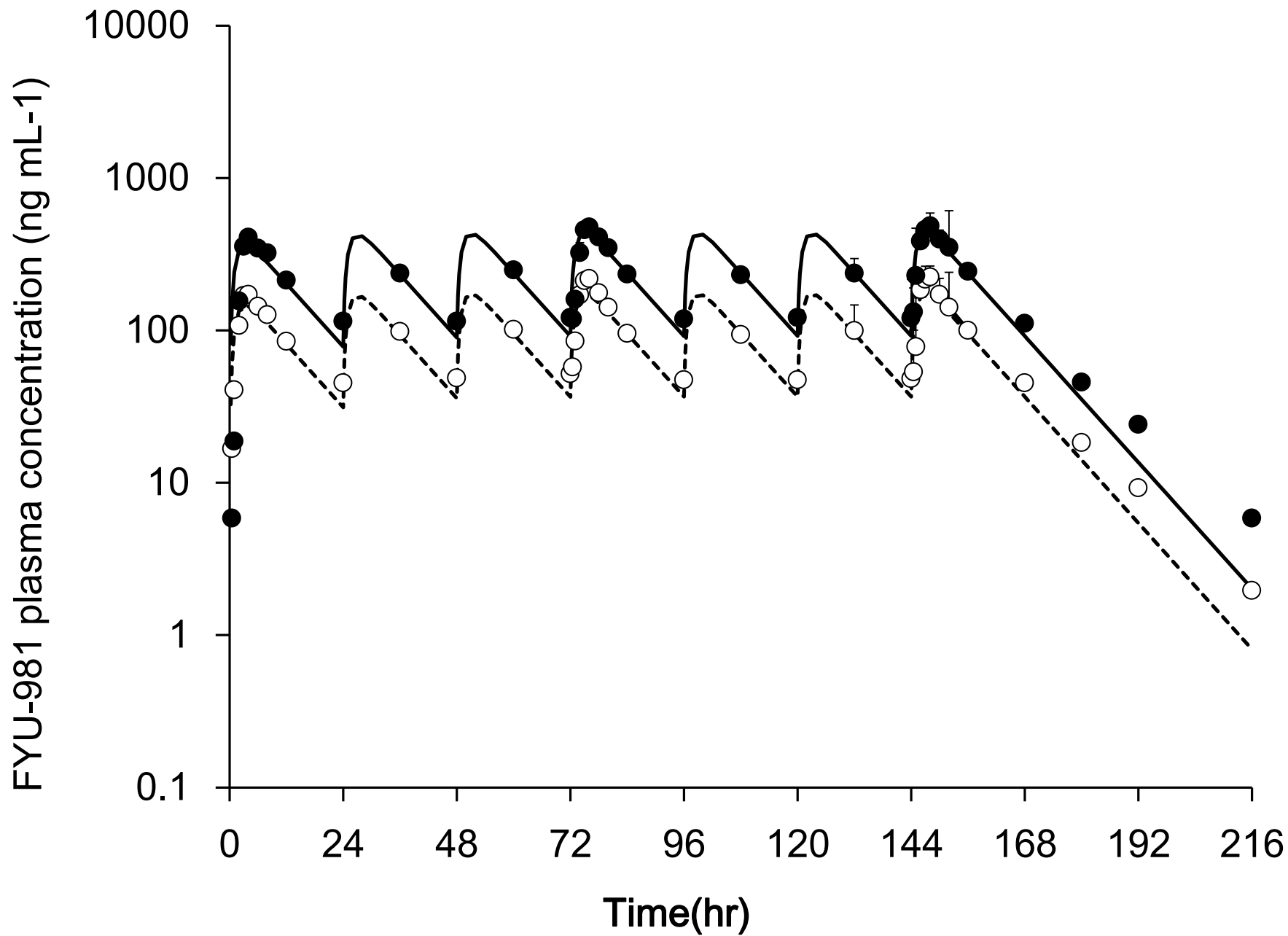

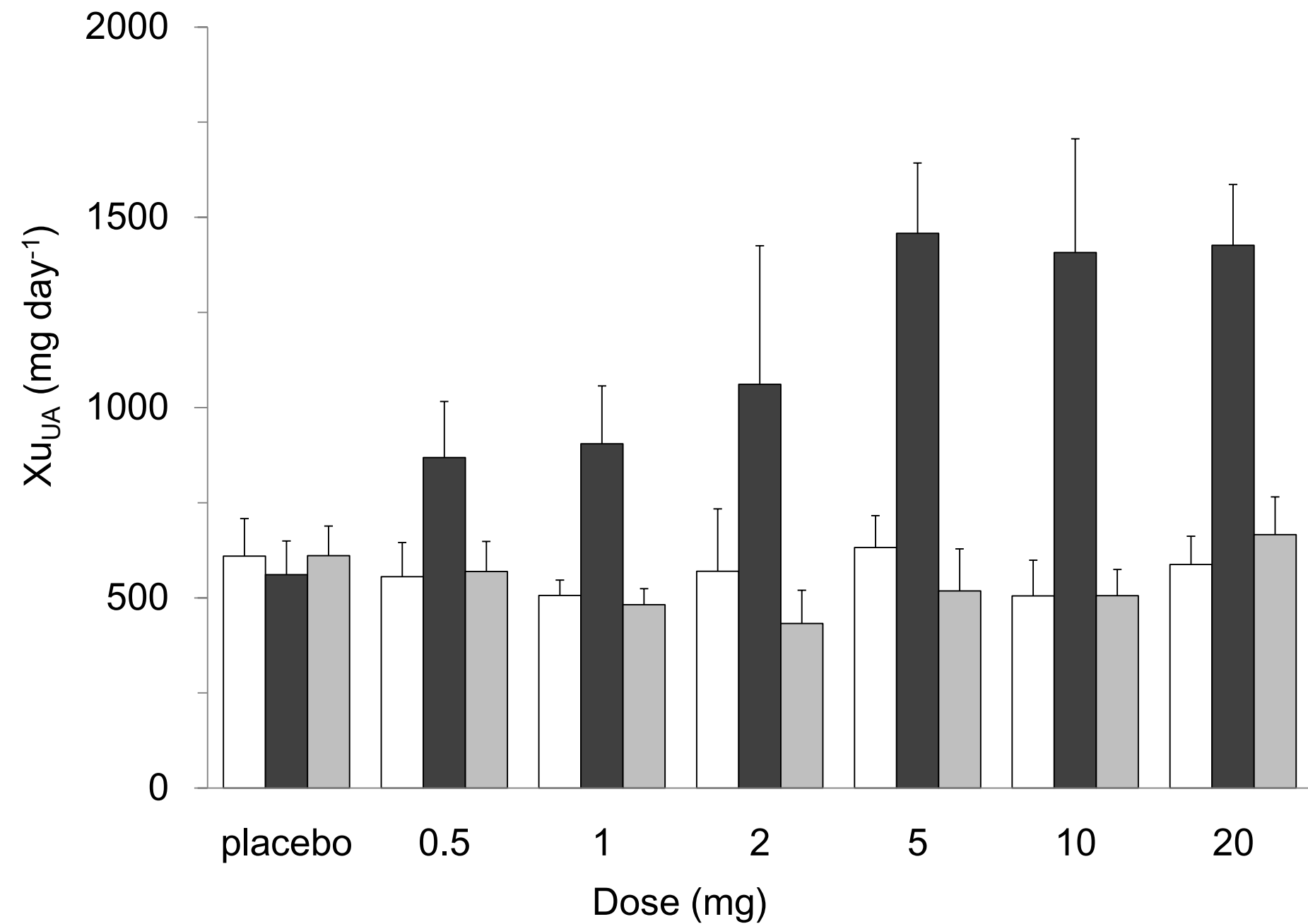

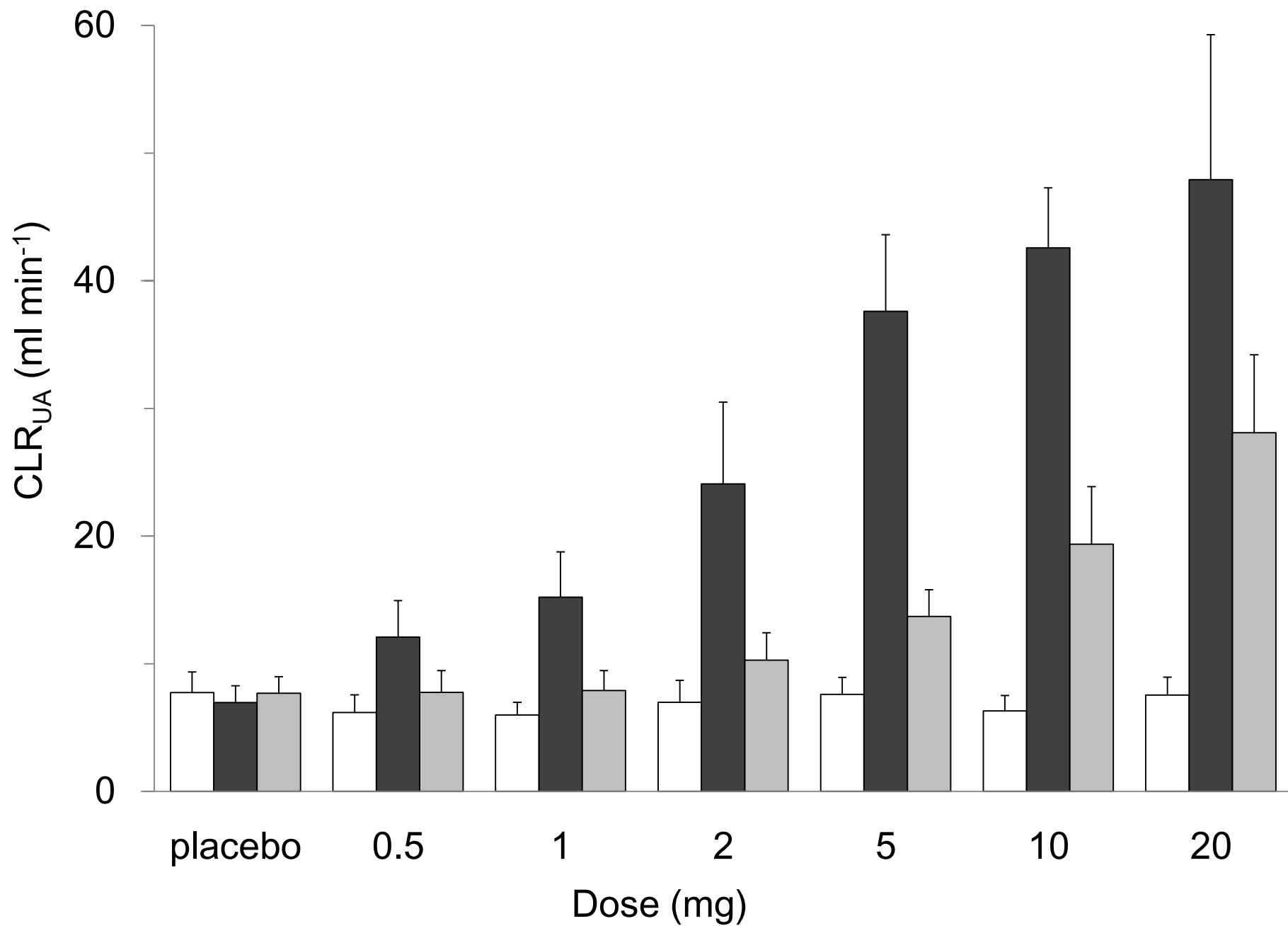

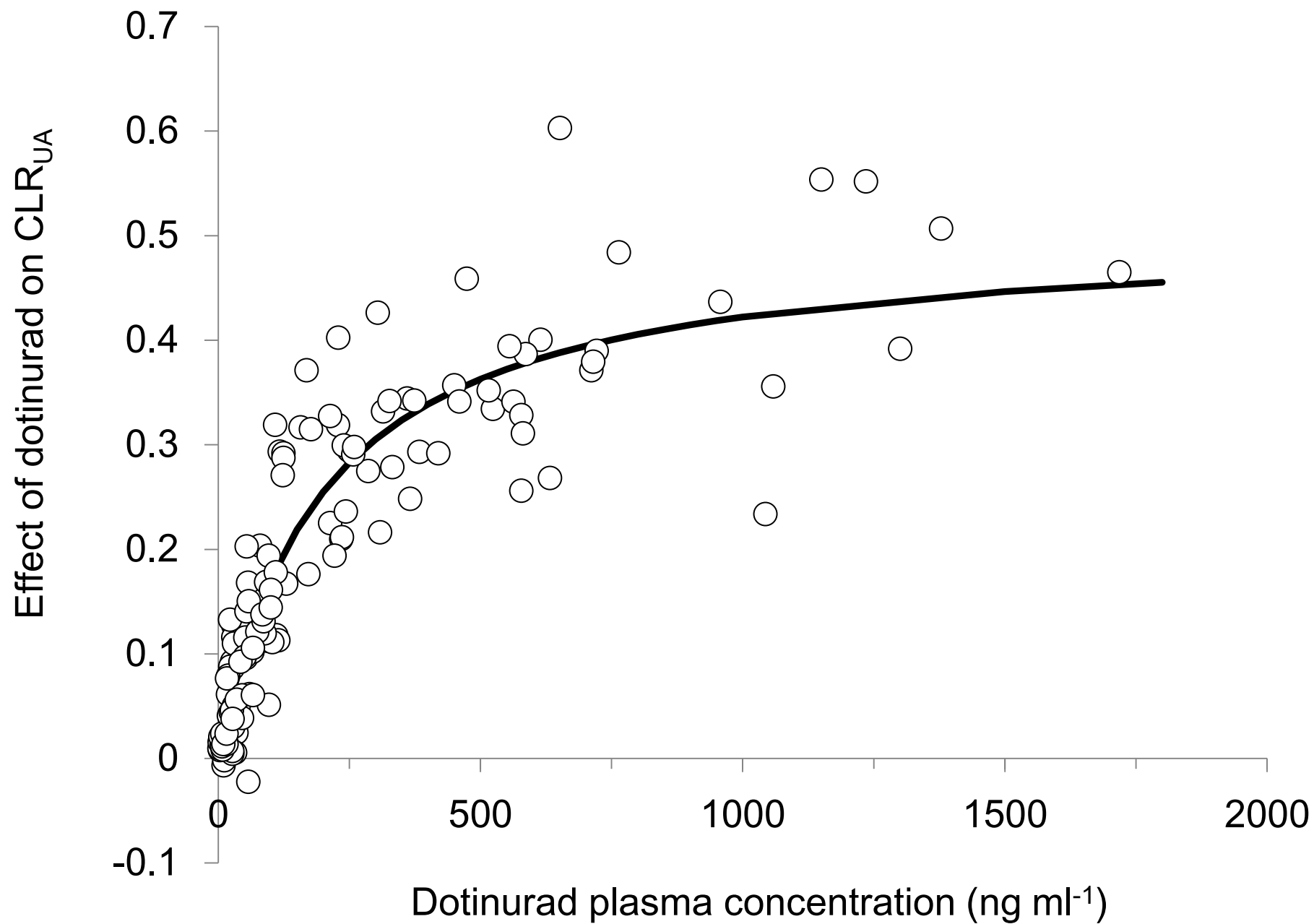

Supplement: Supplementary file 1 [file PRP2-7-e00533-s001.pdf]
