# Supplementary material for: Pharmacokinetic/pharmacodynamic modeling and simulation of dotinurad, a novel uricosuric agent, in healthy volunteers
Source: Pharmacol Res Perspect. 2019 Nov 26;7(6):e00533. doi: 10.1002/prp2.533 (PMC6880184; doi:10.1002/prp2.533)
Supplement: Supplementary file 2 [file PRP2-7-e00533-s002.docx]

**Title**

Pharmacokinetic/pharmacodynamic modeling and simulation of dotinurad, a novel uricosuric agent, in healthy volunteers

Keisuke Motoki^1^, Takako Igarashi^1^, Koichi Omura^1^, Hiroshi Nakatani^2^, Takashi Iwanaga^1^, Ikumi Tamai^3^ and Tetsuo Ohashi^1^

^1^ FUJI YAKUHIN CO., LTD., Saitama, Japan

^2^Department of Research, Clinical Trial Center, Kitasato University Kitasato Institute Hospital, Tokyo, Japan

^3^Faculty of Pharmaceutical Sciences, Institute of Medical, Pharmaceutical and Health Sciences, Kanazawa University, Kanazawa, Japan

**Correspondence**

Keisuke Motoki, Research Laboratories 2, FUJI YAKUHIN CO., LTD., 636-1 Iida-Shinden, Nishi Ward, Saitama City, Saitama, 331-0068, Japan

Tel: +81-48-620-1611. Fax: +81-48-620-1617.

E-mail address: k-motoki@fujiyakuhin.co.jp

**Supporting Information**

Figure S1

The observed and predicted dotinurad plasma concentration-time profiles after multiple dose administrations of 2- or 5-mg dose of dotinurad.

Open symbol and dashed line represents the observed and predicted data at 2 mg. points. Filled symbol and solid line represents the observed and predicted data at 2 mg. points.

Figure S2

Urinary excretion (A) and renal clearance (B) of urate (mean + SD) during 24 h before and after single-dose administration of placebo or 0.5–20-mg dose of dotinurad.

Open bar, -24 to 0 h (baseline); closed bar, 0–24 h; gray bar, 24–48 h.

Figure S3

Pharmacodynamic parameter estimation of the effect of dotinurad on CLR_UA_ by simple E_max_ model analysis.

Each symbol represents the observed effect of dotinurad on CLR_UA_ during 0–6, 6–12, 12–24, and 24–48 h after single-dose administration of each dose of dotinurad. Solid line represents the calculated values using estimated parameters.
